# A Case Report of Suicide Attempt Caused by Acute and Transient Psychotic Disorder during the COVID-19 Outbreak

**DOI:** 10.1155/2020/4320647

**Published:** 2020-05-27

**Authors:** Kai Zhang, Yudong Shi, Huanzhong Liu, Kenji Hashimoto

**Affiliations:** ^1^Department of Psychiatry, Chaohu Hospital of Anhui Medical University, Hefei 238000, China; ^2^Anhui Psychiatric Center, Anhui Medical University, Hefei 238000, China; ^3^Division of Clinical Neuroscience, Chiba University Center for Forensic Mental Health, Chiba 260-8670, Japan

## Abstract

We reported a case of suicide attempt caused by acute and transient psychotic disorder during the COVID-19 outbreak, which broke out in December 2019 in Wuhan. An epidemic of infectious diseases brought great psychological pressure to the public. During this period, a 20-year-old man went to the hospital repeatedly because he suspected that he was infected, with suspicious auditory hallucinations, self-laughter, primary delusions, victimization delusions, relationship delusions, and suicide attempts. He was diagnosed with Acute Transient Psychotic Disorder. 0.1 g bid Quetiapine was given orally, then gradually increased to 0.4 g per day, supplemented by cognitive therapy. The patient was discharged from hospital in relief of symptoms on February 9th. *Conclusion*. During the epidemic period, in addition to strengthening the protection work, we should also monitor the mental and psychological state of the population to prevent mental illness caused by coronavirus.

## 1. Introduction

In December 2019, an unexplained epidemic broke out in Wuhan, Hubei Province, China [1]. A new coronavirus was identified as the pathogen and was later named COVID-19 by the World Health Organization. As of March 13, 2020, there are 81,003 cases of the COVID-19, and 3181 deaths have been confirmed in China, with a mortality rate of about 3.93% [2]. The epidemic not only brings the risk of death after virus infection to the people, but also brings people unbearable psychological pressure. In the face of the extraordinary situation of public health emergencies, people are prone to a variety of psychological and psychological problems [3, 4]. Below, we report a case of suicide caused by acute and transient psychotic disorder (ATPD) during the COVID-19 outbreak.

## 2. Case Report

Mister X, a 20-year-old male college student, living in Hefei, Anhui Province, China. Mister X traveled in Wuhan from January 15 to 18, 2020, and returned to his home in Hefei on January 18, 2020. The COVID-19 epidemic began to break out around January 20. Because he felt feverish and suspected that he was infected with COVID-19, Mister X suddenly went insane at the evening of January 29. Mister X stayed up all night, talking nonsense, sometimes dancing, crying, and laughing. His family sent him to a designated COVID-19 hospital in Hefei on January 29. His body temperature was 37.2°C. The pharynx swab was taken for novel coronavirus nucleic acid test. The chest computed tomography (CT) results showed “left lung nodule”. Outpatient doctors diagnosed “upper respiratory tract infection” without considering COVID-19's diagnosis. But Mister X firmly believed that he had got the COVID-19 and did not believe the doctor's diagnosis. At noon on the same day, he went with his family to a fever clinic of another hospital. During the queuing period at the outpatient clinic, the patient suddenly got emotional and gibberish, saying, “he has been hurt by others”, “Why does someone else not get pneumonia, only he has it? Someone must be trying to hurt him.” The patient used Wechat to tell all his friends that he had COVID-19.

On the morning of the 31st, the patient and his family went to the hospital to get the nucleic acid test report. The patient's nucleic acid test result was negative, but he still cried and laughed, and the family went to the pharmacy to buy antipyretic drugs and azithromycin and other drugs for the patients to take, the symptoms did not improve. At noon on February 1, the patient began not to talk to his family, did not eat or drink, and his expression was painful. Sometimes he yelled and cursed and kept spitting things out of his mouth. The patient suddenly jumped from the sixth floor of his home at around 17 : 00. Luckily, because of the canopy below, he suffered no serious injuries. He was sent to another designated hospital in Hefei for treatment. The CT examination of Mister X showed that “there were no traumatic lesions in the skull, chest and abdomen”, and no obvious fracture was found in the whole body. The patient had a skin wound on the inside of his right ankle that had been sutured, and a skin scratch on his left chest, which was disinfected by iodophor.

On the morning of February 2nd, according to the admission procedure of the psychiatric department of our hospital during the epidemic situation of novel coronavirus, the patient was admitted to a hospital. The results of the peripheral blood test showed that creatine kinase (CK) 1236 U/L was higher than normal, but no obvious abnormality was found in the rest. Both novel coronavirus nucleic acid tests showed negative results. The results of chest CT showed “nodular foci of the left lung”. The diagnosis of novel coronavirus pneumonia was excluded after consultation in the respiratory and infection department. The patient was later admitted to the psychiatric isolation ward of our hospital. ATPD was diagnosed by psychiatric consultation, and quetiapine fumarate was given with an oral rehydration solution of 0.1 g bid per day. After entering the psychiatric department, the blood biochemical examination showed (CK) 1236 U/L, total bilirubin (TB) 46.8 mmol/L, and aspartate aminotransferase (AST) 62 U/L. Routine blood test showed that the percentage of lymphocytes was 18.10%, the percentage of monocytes was 12.10%, the ratio of eosinophils was 7.10%, and the rest was normal. Electrocardiogram (ECG), electroencephalogram (EEG), and cranial CT were normal. The score of Positive and Negative Syndrome Scale (PANSS) was 76, Hamilton Depression Rating Scale (HAMD) was 29, and Hamilton Anxiety Rating Scale (HAMA) was 19. No previous family psychiatric history and no history of drugs. Later, quetiapine gradually increased to 0.4 g per day for oral treatment, supplemented by cognitive therapy. After a week of medication and psychotherapy, the patient's condition was improved, the diet was normal, and the sleep was better than before. Psychiatric symptoms also improved markedly. The score reduction rate of PANSS was more than 50%. The patient is exposed to cooperation and occasionally shows signs of tension, hallucinations, and delusions were not elicited. The patient was discharged from the hospital on February 9.

## 3. Discussion

ATPD is a kind of psychotic syndrome with acute attack and a short course of disease [5]. The patient in this case had a normal mental state in the past, because of the history of contact with epidemic areas, acute onset after the outbreak of the epidemic and fever symptoms, delusions, hallucinations, self-laughter, running abroad, and self-abandonment behavior, which met the diagnostic criteria of ATPD [6]. It has been reported that acute stress events often lead to self-abandonment in patients with ATPD [7]. In this case, patients have jumped from the sixth floor. ATPD can be treated with antipsychotics for a short time [8]. However, long-term use of antipsychotics will lead to metabolic disorders in patients with ATPD, reduce life expectancy, and bring many side effects, so it is not suitable to use these drugs for a long time [9]. Combined with psychotherapy, it has a good effect on the rehabilitation of patients with ATPD. After treatment, the patients with ATPD can be relieved completely and their individual function can be restored to the predisease level. In this case, the patient was treated with antipsychotic quetiapine and supplemented by psychotherapy and energy support therapy. After one week of treatment, the patient's condition was significantly improved. Half a month later, the follow-up results showed that the patient recovered as usual.

In conclusion, we reported the clinical manifestations and changes of ATPD caused by novel coronavirus. This suggests that during the epidemic prevention period of novel coronavirus, in addition to strengthening the protection work, and the treatment of patients, we also need to evaluate people's mental psychology and establish effective psychological counseling channels to reduce the adverse impact of the epidemic on society.
